# TcoFBase: a comprehensive database for decoding the regulatory transcription co-factors in human and mouse

**DOI:** 10.1093/nar/gkab950

**Published:** 2021-10-30

**Authors:** Yuexin Zhang, Chao Song, Yimeng Zhang, Yuezhu Wang, Chenchen Feng, Jiaxin Chen, Ling Wei, Qi Pan, Desi Shang, Yanbing Zhu, Jiang Zhu, Shuangsang Fang, Jun Zhao, Yongsan Yang, Xilong Zhao, Xiaozheng Xu, Qiuyu Wang, Jincheng Guo, Chunquan Li

**Affiliations:** The First Affiliated Hospital, Institute of Cardiovascular Disease, Hengyang Medical School, University of South China, Hengyang, Hunan 421001, China; School of Medical Informatics, Daqing Campus, Harbin Medical University, Daqing 163319, China; The First Affiliated Hospital, Institute of Cardiovascular Disease, Hengyang Medical School, University of South China, Hengyang, Hunan 421001, China; School of Medical Informatics, Daqing Campus, Harbin Medical University, Daqing 163319, China; School of Medical Informatics, Daqing Campus, Harbin Medical University, Daqing 163319, China; The Key Laboratory of Molecular Biology for High Cancer Incidence Coastal Chaoshan Area, Shantou University Medical College, Shantou 515041, China; The First Affiliated Hospital, Institute of Cardiovascular Disease, Hengyang Medical School, University of South China, Hengyang, Hunan 421001, China; School of Medical Informatics, Daqing Campus, Harbin Medical University, Daqing 163319, China; School of Medical Informatics, Daqing Campus, Harbin Medical University, Daqing 163319, China; School of Medical Informatics, Daqing Campus, Harbin Medical University, Daqing 163319, China; School of Medical Informatics, Daqing Campus, Harbin Medical University, Daqing 163319, China; School of Medical Informatics, Daqing Campus, Harbin Medical University, Daqing 163319, China; The First Affiliated Hospital, Institute of Cardiovascular Disease, Hengyang Medical School, University of South China, Hengyang, Hunan 421001, China; School of Computer, University of South China, Hengyang, Hunan 421001, China; The First Affiliated Hospital, Cardiovascular Lab of Big Data and Imaging Artificial Intelligence, Hengyang Medical School, University of South China, Hengyang, Hunan 421001, China; Hunan Provincial Base for Scientific and Technological Innovation Cooperation, University of South China, Hengyang, Hunan 421001, China; Experimental and Translational Research Center, Beijing Friendship Hospital, Capital Medical University, Beijing 100050, China; School of Medical Informatics, Daqing Campus, Harbin Medical University, Daqing 163319, China; Traditional Chinese Medicine, Beijing University of Chinese Medicine, Beijing 100029, China; School of Medical Informatics, Daqing Campus, Harbin Medical University, Daqing 163319, China; School of Medical Informatics, Daqing Campus, Harbin Medical University, Daqing 163319, China; School of Medical Informatics, Daqing Campus, Harbin Medical University, Daqing 163319, China; School of Medical Informatics, Daqing Campus, Harbin Medical University, Daqing 163319, China; The First Affiliated Hospital, Institute of Cardiovascular Disease, Hengyang Medical School, University of South China, Hengyang, Hunan 421001, China; School of Medical Informatics, Daqing Campus, Harbin Medical University, Daqing 163319, China; School of Computer, University of South China, Hengyang, Hunan 421001, China; The First Affiliated Hospital, Cardiovascular Lab of Big Data and Imaging Artificial Intelligence, Hengyang Medical School, University of South China, Hengyang, Hunan 421001, China; Hunan Provincial Base for Scientific and Technological Innovation Cooperation, University of South China, Hengyang, Hunan 421001, China; Traditional Chinese Medicine, Beijing University of Chinese Medicine, Beijing 100029, China; The First Affiliated Hospital, Institute of Cardiovascular Disease, Hengyang Medical School, University of South China, Hengyang, Hunan 421001, China; School of Medical Informatics, Daqing Campus, Harbin Medical University, Daqing 163319, China; School of Computer, University of South China, Hengyang, Hunan 421001, China; The First Affiliated Hospital, Cardiovascular Lab of Big Data and Imaging Artificial Intelligence, Hengyang Medical School, University of South China, Hengyang, Hunan 421001, China; Hunan Provincial Base for Scientific and Technological Innovation Cooperation, University of South China, Hengyang, Hunan 421001, China

## Abstract

Transcription co-factors (TcoFs) play crucial roles in gene expression regulation by communicating regulatory cues from enhancers to promoters. With the rapid accumulation of TcoF associated chromatin immunoprecipitation sequencing (ChIP-seq) data, the comprehensive collection and integrative analyses of these data are urgently required. Here, we developed the TcoFBase database (http://tcof.liclab.net/TcoFbase), which aimed to document a large number of available resources for mammalian TcoFs and provided annotations and enrichment analyses of TcoFs. TcoFBase curated 2322 TcoFs and 6759 TcoFs associated ChIP-seq data from over 500 tissues/cell types in human and mouse. Importantly, TcoFBase provided detailed and abundant (epi) genetic annotations of ChIP-seq based TcoF binding regions. Furthermore, TcoFBase supported regulatory annotation information and various functional annotations for TcoFs. Meanwhile, TcoFBase embedded five types of TcoF regulatory analyses for users, including TcoF gene set enrichment, TcoF binding genomic region annotation, TcoF regulatory network analysis, TcoF-TF co-occupancy analysis and TcoF regulatory axis analysis. TcoFBase was designed to be a useful resource that will help reveal the potential biological effects of TcoFs and elucidate TcoF-related regulatory mechanisms.

## INTRODUCTION

The transcriptional regulation program that establishes and maintains specific cell states in mammals is extremely complicated (1). Studies have revealed that major regulators of the transcriptional regulation program are transcription factors (TFs), transcription co-factors (TcoFs) and chromatin regulators. TFs typically bind in a cooperative fashion to distal DNA elements to regulate gene expression by recruiting interactive TcoFs and forming physical contact loops (2). Recent studies have also provided new insights into TcoFs, which are crucial for DNA loop maintenance and proper gene control. TcoFs play a decisive role in gene expression through interaction with TFs, although they do not have DNA-binding properties of their own. Increasing evidence has demonstrated that dysfunction of TcoFs can lead to aberrant gene expression and cause a broad range of diseases, suggesting that TcoFs may serve as potential therapeutic targets (3–8). Thus, it is urgent to investigate and unveil TcoF-mediated regulatory mechanisms. Importantly, as a type of transcriptional regulator, protein activities of TcoFs can affect the expression of downstream target genes by indirectly occupying DNA regulatory elements, such as promoter regions, enhancer regions and super-enhancer regions. These specific occupancies have an important function in maintaining cell identity. For example, the TcoF Bromodomain Containing 4 (BRD4) recruits many enhancers and super-enhancers, while its inhibition can suppress the transcription level of super-enhancers and enhancers associated genes (9,10). In addition, some studies have also found that upstream signaling perturbations further influence TcoFs and alter the expression levels of downstream target genes. These studies indicated the importance and widespread utility of TcoF-mediated regulatory axes for addressing key issues associated with physiological and pathological processes.

Previous studies have developed several TcoF-related databases to investigate the biological content of TcoFs, which have provided valuable information for decoding the biological functions of TcoFs. For example, TcoFDB (11) stores a list of TcoFs in mammals and supplies information needed to explore the roles of TcoFs. Some TF-related databases also provide useful data for TcoFs, such as AnimalTFDB (12), and ApicoTFdb (13), which offer basic annotation information for TcoFs. Meanwhile, hTFtarget (14) is a comprehensive resource which focused on predicting TcoF–target interactions from a huge set of TcoF ChIP-seq data. However, until now, a comprehensive database to depict comprehensive regulatory elements of TcoFs has not been available. Given the development of high-throughput techniques, chromatin immunoprecipitation sequencing (ChIP-seq) has become an important strategy for identifying the target genes and functions of TcoFs. With the rapid accumulation of TcoF-associated ChIP-seq data, the comprehensive collection and integrative analysis of these data has become an urgent need. More importantly, integrative annotations and analyses will be very useful for elucidating the mechanisms of TcoFs which underlie transcriptional regulation. Therefore, it is highly desirable to construct a comprehensive resource of TcoFs, which provides the extensive annotations of TcoFs and associated regulatory analyses.

**Figure 1.**
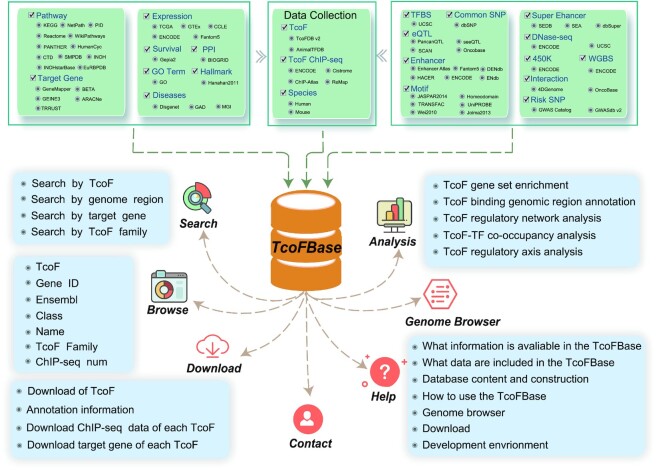
Database content and construction. TcoFBase has a large number of TcoF-related ChIP-seq datasets and abundant curated multi-omics annotations for TcoFs. TcoFBase also contains analytical tools and multiple functions to browse, search, download and visualize TcoFs.

To address this needs, here we developed the TcoFBase database (http://tcof.liclab.net/TcoFbase), which aimed to document a large number of available resources for mammalians TcoFs, and provided extensive annotations and analyses of TcoFs. The current version of TcoFBase curated a total of 2322 TcoFs and 6759 TcoFs associated ChIP-seq data from over 500 tissues and cell types in humans and mice. Emphatically, TcoFBase provided the detailed and abundant (epi) genetic annotations in ChIP-seq-based TcoF binding regions by processing and integrating large scale high throughput experimental data and low throughput experimental data, including super-enhancers, enhancers, transcription factor binding sites (TFBS), methylation sites, common single nucleotide polymorphisms (SNPs), risk SNPs, expression quantitative trait locus (eQTL) and 3D chromatin interactions. TcoFBase also provided TcoF downstream target genes by mapping binding regions onto genomes using five methods. Furthermore, TcoFBase supported upstream regulatory information for TcoFs and various functional annotations, including pathways, gene ontology (GO) terms, cancer hallmarks, protein-protein interactions (PPIs), survival, expression and disease information. In particular, TcoFBase embedded five types of TcoF regulatory analyses for users, including TcoF gene set enrichment, TcoF binding genomic region annotation, TcoF regulatory network analysis, TcoF-TF co-occupancy analysis and TcoF regulatory axis analysis. TcoFBase was also a user friendly database to query, browse, analyze and visualize information associated with TcoFs (Figure 1). We believe that TcoFBase may become a useful and effective platform for exploring potential functions and regulation of TcoFs in diseases and biological processes.

## MATERIALS AND METHODS

### TcoF datasets

A list of TcoFs in mammals was collected from TcoF-DB v2 (11) and AnimalTFDB 3.0 (12) (Figure 1 middle-top panel). Meanwhile, we collected 6759 TcoF-related ChIP-seq datasets in different human and mouse cell and tissue types for human and mouse from ReMap (15), ENCODE (16), Cistrome (17), and ChIP-Atlas (18) (Figure 1, middle-top panel). To standardize the format and genome version, genomic peaks in all ChIP-seq data were converted to the hg19 (human) and mm10 (mouse) genomic versions using the liftOver tool of UCSC (http://genome.ucsc.edu/cgi-bin/hgLiftOver) (19). Additionally, we also provided online liftOver tools to convert genome coordinates for users. Users can substitute hg38 assembly information for hg19 genomic regions and mm10 assembly information for mm39 genomic regions.

### Annotations of TcoF regulatory regions

#### Super-enhancers and enhancers

To annotate the potential roles of TcoF-associated regions, we obtained enhancer data from EnhancerAtlas (20), FAMTOM5 (21), ENCODE (16), HANCER (22), DENdb (23) and ENdb (24), including 14 797 266 human enhancers and 439 092 mouse enhancers (Figure 1 upper right panel and Supplementary Table S1). Some databases identified enhancers by high-throughput experimental data, such as FAMTOM5 (21) and ENCODE (16). Moreover, experimentally validated enhancers were downloaded from ENdb, which were manually curated based on low-throughput experimental data. Moreover, we collected H3K27ac ChIP-seq data from Roadmap, Genomics of Gene Regulation Project, NCBI GEO/SRA and ENCODE (25), which contained both H3K27ac ChIP-seq and the corresponding input controls. We ran Bowtie (26) for each H3K27ac ChIP-seq dataset and all peaks were called using MACS (27). ROSE (10) was used to identify super-enhancer regions. In addition, we also downloaded human and mouse super-enhancers from SEA (28) and dbSuper (29) (Figure 1, upper right panel and Supplementary Table S1). In total, TcoFBase curated 2 678 273 human super-enhancers and 11,609 mouse super-enhancers.

#### TFBS

Studies have revealed that TcoFs contribute to gene transcription by interacting with TFs rather than by directly binding DNA elements. To investigate the co-operative relationships between TFs and TcoFs, we first used Find Individual Motif Occurrences (FIMO) software (30) to identify the motif occurrences in TcoF binding regions. Over 3000 DNA binding motifs for ∼700 mammalian TFs were collected from Jolma2013 (31), JASPAR CORE 2014 vertebrates (32), Homeodomains (33), UniPROBE (34) and Wei2010 (35) (Figure 1 upper right panel and Supplementary Table S1). Motif-based TF binding sites within TcoF binding regions were identified with a *P*-value threshold of 1e–6. Second, we obtained 5 547 656 human TFBS and 2 858 356 mouse TFBS from UCSC (19) and used BEDTools (36) to identify the intersected TF peaks within TcoFs peaks as TFBS (Figure 1 upper right panel and Supplementary Table S1).

#### SNP/Linkage disequilibrium (LD) SNPs/risk SNP/eQTL

To annotate the effects of SNPs in TcoF regulatory regions, we downloaded 38 063 729 human common SNPs from dbSNP (37) and used VCFTools v0.1.13 (38) to calculate SNPs with a minimum allelic frequency (MAF) > 0.05 (Figure 1 upper right panel and Supplementary Table S1). Plink v1.9 (39) was used to identify the LD SNPs (*r*^2^ = 0.8) in five super-populations (East Asian, African, European, Ad Mixed American and South Asian). A total of 264 514 human risk SNPs were downloaded from the GWAS Catalog (40) and GWASdb v2 (41) and 2 886 133 human eQTLs were collected from PancanQTL (42), seeQTL (43), SCAN (44) and Oncobase (45) (Figure 1, upper right panel and Supplementary Table S1).

#### Chromatin interaction/ DNase I hypersensitive signals (DHSs)

Chromatin interaction data (e.g. ChIA-PET 3C, 4C, 5C and Hi-C) are useful for understanding the precise regulatory mechanism between TcoFs and downstream genes. We obtained these data from 4DGenome (46) and Oncobase (45) (Figure 1 upper right panel and Supplementary Table S1). DHS annotation data of TcoF regulatory regions were collected from UCSC (19) and ENCODE (16) for 69 860 705 human DHSs from 293 samples and 9 802 229 mouse DHSs from 56 samples (Figure 1, upper right panel and Supplementary Table S1).

#### Methylation

Methylation data such as 450K arrays and whole-genome shotgun bisulfite sequencing (WGBS) are useful to understand the regulatory relationships between the regulatory regions and TcoFs. We downloaded 30 392 523 methylation sites from 450k arrays and 166 855 665 WGBS datasets from ENCODE (16) (Figure 1, upper right panel and Supplementary Table S1).

### TcoFs target genes and TcoF–target network

We provided five different methods to identify the downstream target genes of TcoFs. First, we used a python script from ROSE (ROSE geneMapper.py) (10) to annotate TcoF downstream target genes in each human and mouse TcoF ChIP-seq dataset (Figure 1, upper left panel). Notably, target genes obtained using three strategies of ROSE (10) (overlap, proximal and closest) were combined and collected. Second, binding and expression target analysis (BETA) (47) was used to identify TcoF downstream genes (Figure 1, upper left panel). BETA is a software package that integrates ChIP-seq data of chromatin regulators to infer direct target genes. BETA minus (47) was used here to identify target genes by inputting bed format files of human and mouse TcoF ChIP-seq datasets. Third, we performed GENIE3 (48) to identify human TcoF target genes by inputting The Cancer Genome Atlas (TCGA) cancer expression matrices (mouse TcoFs target genes were identified by inputting FANTOM5 mouse cell expression matrices) (Figure 1, upper left panel). GENIE3 (48) is a random forest-based approach to predict the strength of putative regulatory links between target genes and their putative regulators. Fourth, we utilized the mutual information-based software ARACNe (49) to identify human TcoF target genes via TCGA expression data (Figure 1, upper left panel). Finally, we identified mammalian TcoF target genes by collecting curated TcoF–target gene pairs from TRRUST v2 (50) (Figure 1, upper left panel). Importantly, TRRUST v2 provided literature-curated TcoF–target pairs in humans and mice. All of the TcoF target genes were embedded in the current version of TcoFBase. We also defined the target gene weight for each TcoF based on calculating the sum of prediction methods. Users can perform hypergeometric test between genes of interest and TcoF target genes to identify the upstream TcoFs regulating the genes. The enrichment significance *P*-value for the particular TcoF was calculated as:

$$\begin{equation*} P=1-\sum_{i-0}^{x-1}\frac{\binom{k}{i}\binom{n-k}{s-i}}{\binom{n}{s}} \end{equation*}$$

where *n* is the number of TcoF target genes and *k* is the number of input genes, of which *x* genes are involved in the TcoF containing s target genes.

Importantly, we constructed a human TcoF–target network by merging the TcoF–target genes from the above five methods. The mouse TcoF–target network was constructed by merging the TcoF–target genes from the above four methods except for ARACNe. To ensure the high credibility of the network, we reserved TcoF–target pairs that were supported by more than half of the methods. For human, we reserved the TcoF-gene pairs that were identified in more than three methods. For mice, TcoF-gene pairs that were identified in more than two methods were reserved. Based on this network, users can locate the sub-network of interest to find the TcoF mediated regulatory axes.

### Functional annotations of TcoFs

To better illustrate the biological functions of TcoFs, we collected more annotation information of TcoFs, including TcoF associated pathways, gene ontologies, hallmarks, survival, expression and diseases from multiple sources. Briefly, we collected expression matrices with the FPKM value of human TcoFs from TCGA (51), GTEx (52), CCLE (53) and ENCODE (16) (Figure 1, upper left panel). Moreover, TcoFBase provides the TPM value of mouse TcoFs from FANTOM5 (54) and the FPKM value of mouse TcoFs from ENCODE (Figure 1, upper left panel). The experimentally supported mammalian (human and mouse) TcoF-disease associations were collected from DisGeNET (55), GAD (56) and MGI (57) (Figure 1, upper left panel). Meanwhile, we obtained PPIs from BIOGRID (58). The detailed information for 2,169 human and mouse pathways were integrated from a database of ten pathways, including KEGG, Reactome, PANTHER, SMPDB, NetPath, PID, HumanCyc, CTD, WikiPathways, INOHstarBase v2.0 and EuRBPDB (59–61) (Figure 1, upper left panel). Users can select at least one pathway database when using the online analysis tool of ‘TcoF regulatory axis analysis’. In addition, we obtained ten cancer hallmarks from Hanahan and Weinberg (62) and 31 GO terms from GO (63) (Figure 1, upper left panel). We used a python package from GEPIA2 to obtain TCGA cancer survival maps of TcoFs (Figure 1, upper left panel).

## DATABASE USE AND ACCESS

### A search interface for retrieving TcoFs

**Figure 2.**
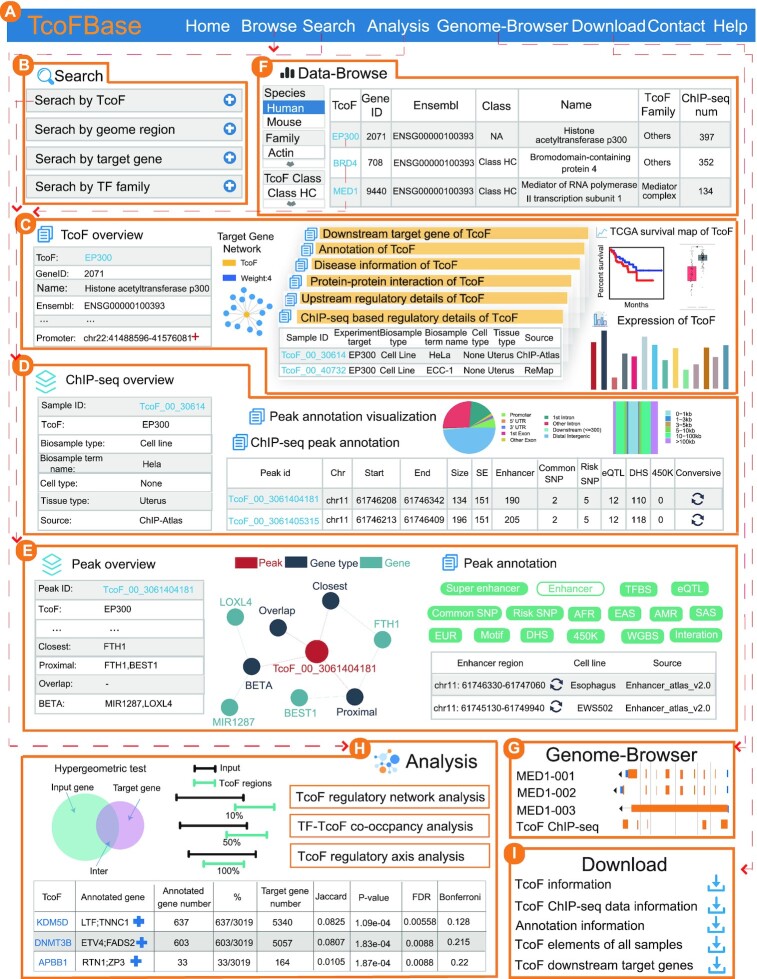
Main functions and usage of TcoFBase. (**A**) The navigation bar of TcoFBase. (**B**) Four inquiry modes are available. (**C**) Search results including TcoF overview, detailed interactive tables of ChIP-seq information, upstream regulatory details, PPIs, disease information, annotation, expression, downstream target genes and TCGA cancer survival maps. (**D**) Interactive table with detailed information about ChIP-seq samples of interest and visualization of peak annotation. (**E**) Detailed interactive tables of annotation information and ChIP-seq peak associated genes are identified through four strategies. Network diagram about these regions is displayed. (**F**) Browsing TcoFs. (**G**) Genome browser. (**H**) An online analysis tools for TcoF. (**I**) Data download.

TcoFBase is a powerful platform that enables users to search, browse, analyze, visualize and download TcoFs of interest (Figure 1, bottom panel). On the ‘Search’ page, TcoFBase provides four query methods for searching TcoF information, including ‘Search by TcoF’ (input a TcoF), ‘Search by genomic region’ (input a genomic region), ‘Search by target gene’ (input a gene official symbol) and ‘Search by TcoF family’ (input a TcoF family name; Figure 2A and B). In the TcoF query, users can input a TcoF, and TcoFBase will return the detailed page of the input TcoF. As a result, users can obtain the details of searched TcoFs, including TcoF Overview, TcoF target gene network, ChIP-seq based regulatory details, downstream target genes, upstream regulatory details, function annotation, PPI, survival, expression and disease information (Figure 2C). In detail, in the table of downstream target genes, TcoFBase provides the downstream target genes of TcoF through five different prediction methods (ROSE, BETA, GENIE3, ARACNe and TRRUST). For upstream regulatory details, the table describes TcoF upstream regulatory regions, including promoter region, enhancer region and super-enhancer region, and the detailed (epi) genetic annotations within regulatory regions are also listed. Users can click ‘Sample ID’ for details about each ChIP-seq sample. TcoFBase displays ChIP-seq overview, ChIP-seq peak annotation and peak annotation visualization. For ChIP-seq peak annotation, the interactive table describes the Peak ID, genome location, size and detailed (epi) genetic information in TcoF ChIP-seq regions (Figure 2D). TcoFBase provides genomic annotations and the distribution of TcoF-binding regions relative to TSS to visualize peaks using ChIPseeker. Moreover, users can click ‘Peak ID’ to obtain detailed information about TcoF ChIP-seq regions. On the peak details page, TcoFBase displays a peak overview, statistics charts and the peak-gene network. The peak-gene network is provided through BETA and ROSE. In ‘Peak annotation’, TcoFBase lists detailed (epi) genetic annotation information, including super-enhancers, enhancers, common SNPs, risk SNPs, eQTLs, DHSs, LD SNPs, TFs predicted by motifs, TFBS, 3D chromatin interactions, methylation sites of the 450k array and WGBS. Meanwhile, we provided the hg38 and mm39 assembly version of each genomic region. By clicking the conversion button, users can find the corresponding hg38 or mm39 versions of these regions. In addition, TcoFBase provides Pearson's correlation coefficients between TcoFs and their target genes in different TCGA cancers (Figure 2E).

In the ‘Search by genomic region’ query, with the input of a genomic region TcoFBase will identify TcoF ChIP-seq peak regions that overlap the submitted region and output a results table. Users can click ‘Peak ID’ to obtain the detailed information about the peak. In the ‘Search by target gene’ query, users can submit a gene of interest and select the target gene prediction methods (ROSE, BETA, GENIE3, ARACNe and TRRUST); TcoFBase will extract its upstream TcoFs in the result table. Users can also select a TcoF family in the ‘Search by TcoF family’ query. TcoFBase can display TcoFs of this family on the results page.

### User-friendly browsing and genome browser

The ‘Browse’ page is organized as an interactive table that allows users to quickly search for TcoFs. Users can use the sorting function and customize filters (‘Species’, ‘Family’ and ‘TcoF Class’) to quickly search TcoFs. To further view the detailed information for TcoFs, users can click the ‘TcoF’ button (Figure 2F). TcoFBase uses the JBrowse genome browser to visualize the regulatory information of human TcoFs by customizing tracks of interest, such as TcoF ChIP-seq regions, gene, enhancer, super-enhancer and DHS (Figure 2G).

### Online analysis tools

We designed five types of analyses to elucidate the mechanistic studies of TcoFs underlying transcriptional regulation (Figure 2H): (I) *TcoF gene set enrichment*. Users can input a gene set of interest, choose the species (human or mouse) and set the *P*-value/FDR to perform TcoF gene set enrichment. TcoFBase will identify upstream TcoF regulators that are significantly enriched by hypergeometric test in the highly credible TcoF–target network. (II) *TcoF binding genomic region annotation*. In this analysis, input of genomic region(s) in bed format will identify the TcoFs that can bind to input genomic region(s). (III) *TcoF regulatory network analysis*. Users can submit a gene list to extract a TcoF-mediated regulatory network from the highly credible TcoF–target network. The TcoF-mediated regulatory network consists of submitted genes and their regulated TcoFs. (IV) *TcoF–TF co-occupancy analysis*. Users can submit a TcoF–TF pair or individual TcoF/TF(s) of interest and choose the species (human or mouse) to identify the TcoF-TF pair(s) with the same DNA binding loci. Users can set different statistical thresholds to control for false positivity by the ‘FIMO’ option. (V) *TcoF regulatory axis analysis*. Users can input gene(s) of interest and users select at least one pathway database. TcoFBase will then identify significantly enriched pathways in which terminal downstream genes are TcoFs.

### Data download

TcoFBase supported download of TcoF-associated files, including ‘TcoF information’, ‘TcoF ChIP-seq data information’, ‘TcoF ChIP-seq samples of different genome versions’ and ‘TcoF downstream target genes’ (Figure 2I). In addition, the database supported the packaged downloads of all TcoF ChIP-seq samples and TcoF downstream target genes. Meanwhile, we also provided download of annotation information, such as PPI, pathway, disease, super-enhancer, enhancer, common SNP, risk SNP, eQTL and DHS. TcoFBase supported export for all data tables in TcoF detail pages, search results and analysis results.

### Case study

#### Case study of BRD4

**Figure 3.**
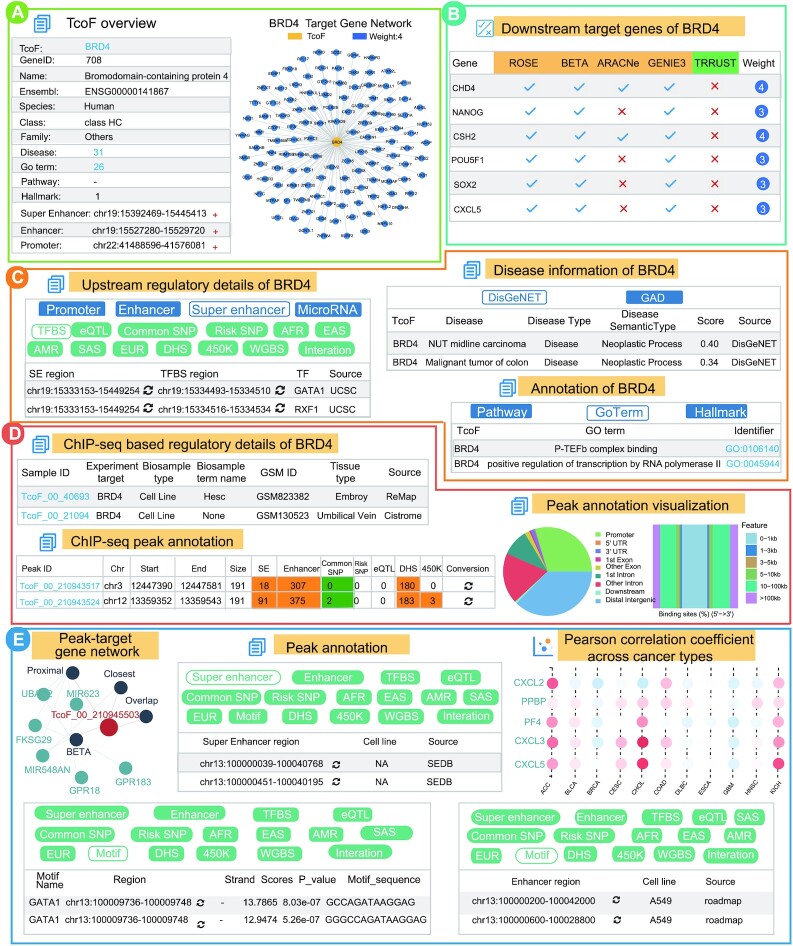
Validation results associated with BRD4. (**A**) BRD4 overview and BRD4 target network. (**B**) Downstream target genes of BRD4. (**C**) Upstream regulatory information, disease and functional annotation of BRD4. (**D**) ChIP-seq information for BRD4, detailed information and visualization of peak annotation about TcoF_00_21094. (**E**) Peak-target gene network, detailed interactive table of annotation information (super-enhancer, motif and enhancer) of TcoF_00_210943517 and Pearson's correlation coefficients between BRD4 and downstream target genes.

To illustrate how to use TcoFBase, we used TcoF ‘BRD4’ as input for ‘Search by TcoF’. BRD4 has been demonstrated to play a crucial role in transcriptional regulation during tumorigenesis and embryogenesis. On the results page, users first obtain the overview information for BRD4, including basic gene annotations, upstream regulatory elements (promoter, enhancer and super-enhancer) and the target gene network (Figure 3A). Here, we also provide the detailed weights of the target genes in the table of ‘Downstream target genes’. Intriguingly, some known target genes of BRD4 such as NANOG, MYOD1 and POU5F1 were identified in this search as highly convincing targets with high weights (64–66), suggesting the potential of using TcoFBase for the identification of TcoF target genes (Figure 3B).

Second, TcoFBase provides upstream regulatory information for BRD4, annotation of BRD4 and disease information for BRD4. In the upstream regulatory aspect, TcoFBase displays abundant (epi) genetics annotations of TcoF upstream regulatory regions. For instance, the TFBS of two TFs, GATA1 and SRF, were found to bind the upstream super-enhancer regions of BRD4 and regulate the expression of BRD4. GATA1 and SRF were previously validated as upstream regulators of BRD4 (Figure 3C upper left table) (67). Importantly, TcoFBase has the strong ability to uncover downstream regulatory clues for BRD4 via processing of BRD4-related ChIP-seq data. Users can click ‘Sample ID’ to obtain the detailed annotations of BRD4 ChIP-seq data (Figure 3D). Notably, as a result of the peak annotation visualization of BRD4 ChIP-seq data, we found that more than half of the BRD4 binding peaks were located in distal regions (>10 kb), which coincides with the regulatory role of BRD4 in super-enhancer mediated transcriptional regulation (Figure 3D right) (68).

Finally, TcoFBase also displays detailed information for each peak of BRD4, such as downstream target genes, downstream regulatory information and Pearson correlation coefficients between BRD4 and downstream target genes across TCGA cancers. For example, CXCL5 was identified as the target gene of BRD4 peak ‘TcoF_00_210943517’ (Figure 3E, upper left). In the peak annotations of ‘TcoF_00_210943517’, we found that SOX2 was occupied at this BRD4 peak, which implied that BRD4 might regulate CXCL5 expression by cooperating with SOX2 (Figure 3E, bottom left). Actually, previous studies have demonstrated the cooperative fashion in which SOX2 and BRD4 regulated CXCL5 expression in glioblastoma (69). BRD4 was considered to be a key regulator in super-enhancer/enhancer formation. TcoFBase also identified a subset of super-enhancers and enhancers that overlapped with peak ‘TcoF_00_210943517’, suggesting that the regulatory mechanisms between BRD4 and CXCL5 were mediated by distal regulatory elements (Figure 3E, middle and bottom right). These results indicated that TcoFBase could be used to explore the potential regulatory mechanisms for TcoFs.

#### Case study of online analysis

In addition, we also provided two case studies of online analysis to verify the function of TcoFBase. We utilized differentially expressed genes of breast invasive carcinoma (BRCA) as inputs for ‘TcoF gene set enrichment’ analysis and the output table of significantly enriched TcoFs was showed in Supplementary Table S2. As a result, EGFR was identified as the top enriched TcoF from the enrichment analysis results, which was demonstrated to play key roles in breast carcinogenesis (Supplementary Materials and Supplementary Figure S1). We also performed the same tests for heart failure in mouse and found that Tnni2 was significantly enriched, which is critical for heart failure (Supplementary Figure S2 and Supplementary Table S3). These results demonstrated the availability and biological value of TcoFBase for TcoF research.

## DISCUSSION

TcoFs have been demonstrated to participate in transcriptional regulation by interacting with TFs and maintaining the DNA contact loops in mammals. Thoroughly revealing TcoF-mediated transcription regulation networks is crucial to understand the molecular mechanisms underlying physiological and pathological processes. Some databases, such as TcoFDB (11), AnimalTFDB (12), hTFtarget (14) and ApicoTFdb (13), have curated the basic knowledge, annotations and predicted target for a large number of TcoFs. However, a comprehensive regulatory database for TcoFs has not been built. Meanwhile, the rapid release of TcoF-associated ChIP-seq data has enabled us to find the target genes of TcoFs and construct a TcoF related global regulatory network. Based on the urgent need and available regulatory data, we developed TcoFBase to document mammalians TcoFs, and provide annotations and enrichment analyses of TcoFs. The current version of TcoFBase has incorporated a total of 2322 TcoFs and 6759 TcoF- associated ChIP-seq data from over 500 tissues and cell types in humans and mice. Table 1 compares TcoFBase with previous TcoF-related databases for TcoF number, TcoF ChIP-seq number and datasets source. As a result, TcoFBase is advanced in multiple aspects, such as TcoF ChIP-seq data scale and abundant (epi) genetic annotations in TcoF binding regions (Table 1 and Supplementary Table S1). Moreover, TcoFBase also supports upstream regulatory information and multiple functional annotations for TcoFs. Overall, TcoFBase is an efficient resource for investigating the regulatory cues of TcoFs.

**Table 1:** . Comparison of TcoF associated information in TcoFBase with other databases

Parameter	TcoFBase	TcoFDBv2	AnimalTFDB3.0	hTFtarget
TcoF (human and mouse) number	2322	1386	1995	Less than 200
TcoF ChIP-seq sample number	6759	–	–	Less than 1000
Region annotation	√	–	–	–
Downstream target genes	√	–	–	√
Peak annotation visualization	√	–	–	–
Genome browser	√	√	–	√
Online analysis	√	–	–	–
GO Term	√	√	√	–
Cancer hallmark	√	–	–	–
Survival	√	–	–	–
PPI	√	√	√	–
Pathway sources	10^a^	2	2	–
Expression sources	5^b^	2	5	–
Disease information sources	3^c^	3	–	–

^a^Pathway were collected from ten resources: KEGG, Reactome, PANTHER, SMPDB, NetPath, PID, HumanCyc, CTD, WikiPathways, INOHstarBase v2.0 and EuRBPDB.

^b^Expression matrixes were collected from five resources: ENCODE, GTEx, CCLE, NCBI and FANTOM5.

^c^Disease were collected from three resources: DisGeNET, GAD and MGI.

As far as we know, TcoFBase is the first integrative resource to focus on unveiling the regulatory mechanisms of TcoFs. Researchers can explore regulatory information about TcoFs and perform TcoF-associated regulatory analyses of interest. TcoFBase has multiple advantages, including (i) comprehensive genetic and epigenetic annotations in TcoF- associated ChIP-seq regions and user-friendly displays on the details page; (ii) identification of TcoF downstream target genes by five methods; (iii) integrative annotation information about TcoF upstream regulatory regions; (iv) useful online analysis tools; (v) four search methods to access TcoFs; (vi) user-friendly browsing and (vii) full-featured detail pages containing disease information, survival and different functional annotations of TcoFs.

The current version of TcoFBase processed a large number of TcoF-related ChIP-seq datasets and curated abundant multi-omics annotations of TcoFs. We compared TcoFBase with other TcoF-associated databases for information and functions, which showed the advantages of TcoFBase. However, we did not use the same methods to detect peaks of TcoFs and we also found that the regulatory information for some TcoFs was not supported by ChIP-seq due to lack of data. In future updates, we will collect ChIP-seq samples of TcoFs to identify TcoF regions. Furthermore, we will extend the scale of the regulatory datasets and add more annotation information. We will also embed more precise methods to infer TcoF-mediated regulatory networks and explore the functions and regulation of TcoFs. In summary, TcoFBase is a user-friendly database to query, browse, analyze and visualize information associated with TcoFs. We believe that TcoFBase will become a useful and effective platform for exploring potential functions and regulation of TcoFs in diseases and biological processes.

## DATA AVAILABILITY

TcoFBase is a comprehensive database which provided annotations and enrichment analyses of TcoFs (http://tcof.liclab.net/TcoFbase). Detailed information is available under the Download page of TcoF.

## Supplementary Material

gkab950_Supplemental_Files

## References

[1] Lee T.I. , Young R.A. Transcriptional regulation and its misregulation in disease. Cell. 2013; 152:1237–1251. 23498934 10.1016/j.cell.2013.02.014 PMC3640494

[2] Zabidi M.A. , Stark A. Regulatory enhancer-core-promoter communication via transcription factors and cofactors. Trends Genet.: TIG. 2016; 32:801–814. 27816209 10.1016/j.tig.2016.10.003 PMC6795546

[3] Tao C.C. , Hsu W.L., Ma Y.L., Cheng S.J., Lee E.H. Epigenetic regulation of HDAC1 SUMOylation as an endogenous neuroprotection against Abeta toxicity in a mouse model of Alzheimer's disease. Cell Death Differ. 2017; 24:597–614. 28186506 10.1038/cdd.2016.161 PMC5384022

[4] Kim S.Y. , Zhang X., Schiattarella G.G., Altamirano F., Ramos T.A.R., French K.M., Jiang N., Szweda P.A., Evers B.M., May H.I. et al. Epigenetic reader BRD4 (Bromodomain-Containing Protein 4) governs nucleus-encoded mitochondrial transcriptome to regulate cardiac function. Circulation. 2020; 142:2356–2370. 33113340 10.1161/CIRCULATIONAHA.120.047239 PMC7736324

[5] Ummarino D. Heart failure: BRD4 inhibition slows HF progression. Nat. Rev. Cardiol. 2017; 14:382–383. 10.1038/nrcardio.2017.86 28569271

[6] Meyer S.N. , Scuoppo C., Vlasevska S., Bal E., Holmes A.B., Holloman M., Garcia-Ibanez L., Nataraj S., Duval R., Vantrimpont T. et al. Unique and shared epigenetic programs of the CREBBP and EP300 acetyltransferases in germinal center B cells reveal targetable dependencies in lymphoma. Immunity. 2019; 51:535–547. 31519498 10.1016/j.immuni.2019.08.006 PMC7362711

[7] Russo J.W. , Nouri M., Balk S.P. androgen receptor interaction with mediator complex is enhanced in castration-resistant prostate cancer by CDK7 phosphorylation of MED1. Cancer Discov. 2019; 9:1490–1492. 31676563 10.1158/2159-8290.CD-19-1028 PMC6830511

[8] Bai L. , Jia Y., Viswakarma N., Huang J., Vluggens A., Wolins N.E., Jafari N., Rao M.S., Borensztajn J., Yang G. et al. Transcription coactivator mediator subunit MED1 is required for the development of fatty liver in the mouse. Hepatology. 2011; 53:1164–1174. 21480322 10.1002/hep.24155 PMC3076129

[9] Tasdemir N. , Banito A., Roe J.S., Alonso-Curbelo D., Camiolo M., Tschaharganeh D.F., Huang C.H., Aksoy O., Bolden J.E., Chen C.C. et al. BRD4 connects enhancer remodeling to senescence immune surveillance. Cancer Discov. 2016; 6:612–629. 27099234 10.1158/2159-8290.CD-16-0217 PMC4893996

[10] Loven J. , Hoke H.A., Lin C.Y., Lau A., Orlando D.A., Vakoc C.R., Bradner J.E., Lee T.I., Young R.A. Selective inhibition of tumor oncogenes by disruption of super-enhancers. Cell. 2013; 153:320–334. 23582323 10.1016/j.cell.2013.03.036 PMC3760967

[11] Schmeier S. , Alam T., Essack M., Bajic V.B. TcoF-DB v2: update of the database of human and mouse transcription co-factors and transcription factor interactions. Nucleic Acids Res. 2017; 45:D145–D150. 27789689 10.1093/nar/gkw1007 PMC5210517

[12] Hu H. , Miao Y.R., Jia L.H., Yu Q.Y., Zhang Q., Guo A.Y. AnimalTFDB 3.0: a comprehensive resource for annotation and prediction of animal transcription factors. Nucleic Acids Res. 2019; 47:D33–D38. 30204897 10.1093/nar/gky822 PMC6323978

[13] Sardar R. , Kaushik A., Pandey R., Mohmmed A., Ali S., Gupta D. ApicoTFdb: the comprehensive web repository of apicomplexan transcription factors and transcription-associated co-factors. Database. 2019; 2019:baz094. 31529106 10.1093/database/baz094 PMC6748703

[14] Zhang Q. , Liu W., Zhang H.M., Xie G.Y., Miao Y.R., Xia M., Guo A.Y. hTFtarget: a comprehensive database for regulations of human transcription factors and their targets. Genomics Proteomics Bioinformatics. 2020; 18:120–128. 32858223 10.1016/j.gpb.2019.09.006 PMC7647694

[15] Cheneby J. , Menetrier Z., Mestdagh M., Rosnet T., Douida A., Rhalloussi W., Bergon A., Lopez F., Ballester B. ReMap 2020: a database of regulatory regions from an integrative analysis of Human and Arabidopsis DNA-binding sequencing experiments. Nucleic Acids Res. 2020; 48:D180–D188. 31665499 10.1093/nar/gkz945 PMC7145625

[16] Consortium E.P. An integrated encyclopedia of DNA elements in the human genome. Nature. 2012; 489:57–74. 22955616 10.1038/nature11247 PMC3439153

[17] Zheng R. , Wan C., Mei S., Qin Q., Wu Q., Sun H., Chen C.H., Brown M., Zhang X., Meyer C.A. et al. Cistrome Data Browser: expanded datasets and new tools for gene regulatory analysis. Nucleic Acids Res. 2019; 47:D729–D735. 30462313 10.1093/nar/gky1094 PMC6324081

[18] Oki S. , Ohta T., Shioi G., Hatanaka H., Ogasawara O., Okuda Y., Kawaji H., Nakaki R., Sese J., Meno C. ChIP-Atlas: a data-mining suite powered by full integration of public ChIP-seq data. EMBO Rep. 2018; 19:e46255. 30413482 10.15252/embr.201846255 PMC6280645

[19] Haeussler M. , Zweig A.S., Tyner C., Speir M.L., Rosenbloom K.R., Raney B.J., Lee C.M., Lee B.T., Hinrichs A.S., Gonzalez J.N. et al. The UCSC Genome Browser database: 2019 update. Nucleic Acids Res. 2019; 47:D853–D858. 30407534 10.1093/nar/gky1095 PMC6323953

[20] Gao T. , Qian J. EnhancerAtlas 2.0: an updated resource with enhancer annotation in 586 tissue/cell types across nine species. Nucleic Acids Res. 2020; 48:D58–D64. 31740966 10.1093/nar/gkz980 PMC7145677

[21] Consortium, F., the, R.P., Clst, Forrest, A.R. Kawaji H. , Rehli M., Baillie J.K., de Hoon M.J., Haberle V., Lassmann T. et al. A promoter-level mammalian expression atlas. Nature. 2014; 507:462–470. 24670764 10.1038/nature13182 PMC4529748

[22] Wang J. , Dai X., Berry L.D., Cogan J.D., Liu Q., Shyr Y. HACER: an atlas of human active enhancers to interpret regulatory variants. Nucleic Acids Res. 2019; 47:D106–D112. 30247654 10.1093/nar/gky864 PMC6323890

[23] Ashoor H. , Kleftogiannis D., Radovanovic A., Bajic V.B. DENdb: database of integrated human enhancers. Database : the journal of biological databases and curation. 2015; 2015:bav085. 26342387 10.1093/database/bav085 PMC4560934

[24] Bai X. , Shi S., Ai B., Jiang Y., Liu Y., Han X., Xu M., Pan Q., Wang F., Wang Q. et al. ENdb: a manually curated database of experimentally supported enhancers for human and mouse. Nucleic Acids Res. 2020; 48:D51–D57. 31665430 10.1093/nar/gkz973 PMC7145688

[25] Jiang Y. , Qian F., Bai X., Liu Y., Wang Q., Ai B., Han X., Shi S., Zhang J., Li X. et al. SEdb: a comprehensive human super-enhancer database. Nucleic Acids Res. 2019; 47:D235–D243. 30371817 10.1093/nar/gky1025 PMC6323980

[26] Langmead B. , Trapnell C., Pop M., Salzberg S.L. Ultrafast and memory-efficient alignment of short DNA sequences to the human genome. Genome Biol. 2009; 10:R25. 19261174 10.1186/gb-2009-10-3-r25 PMC2690996

[27] Zhang Y. , Liu T., Meyer C.A., Eeckhoute J., Johnson D.S., Bernstein B.E., Nusbaum C., Myers R.M., Brown M., Li W. et al. Model-based analysis of ChIP-Seq (MACS). Genome Biol. 2008; 9:R137. 18798982 10.1186/gb-2008-9-9-r137 PMC2592715

[28] Chen C. , Zhou D., Gu Y., Wang C., Zhang M., Lin X., Xing J., Wang H., Zhang Y. SEA version 3.0: a comprehensive extension and update of the super-enhancer archive. Nucleic Acids Res. 2020; 48:D198–D203. 31667506 10.1093/nar/gkz1028 PMC7145603

[29] Khan A. , Zhang X. dbSUPER: a database of super-enhancers in mouse and human genome. Nucleic Acids Res. 2016; 44:D164–D171. 26438538 10.1093/nar/gkv1002 PMC4702767

[30] Grant C.E. , Bailey T.L., Noble W.S. FIMO: scanning for occurrences of a given motif. Bioinformatics. 2011; 27:1017–1018. 21330290 10.1093/bioinformatics/btr064 PMC3065696

[31] Jolma A. , Yan J., Whitington T., Toivonen J., Nitta K.R., Rastas P., Morgunova E., Enge M., Taipale M., Wei G. et al. DNA-binding specificities of human transcription factors. Cell. 2013; 152:327–339. 23332764 10.1016/j.cell.2012.12.009

[32] Mathelier A. , Zhao X., Zhang A.W., Parcy F., Worsley-Hunt R., Arenillas D.J., Buchman S., Chen C.Y., Chou A., Ienasescu H. et al. JASPAR 2014: an extensively expanded and updated open-access database of transcription factor binding profiles. Nucleic Acids Res. 2014; 42:D142–D147. 24194598 10.1093/nar/gkt997 PMC3965086

[33] Berger M.F. , Badis G., Gehrke A.R., Talukder S., Philippakis A.A., Pena-Castillo L., Alleyne T.M., Mnaimneh S., Botvinnik O.B., Chan E.T. et al. Variation in homeodomain DNA binding revealed by high-resolution analysis of sequence preferences. Cell. 2008; 133:1266–1276. 18585359 10.1016/j.cell.2008.05.024 PMC2531161

[34] Robasky K. , Bulyk M.L. UniPROBE, update 2011: expanded content and search tools in the online database of protein-binding microarray data on protein-DNA interactions. Nucleic Acids Res. 2011; 39:D124–D128. 21037262 10.1093/nar/gkq992 PMC3013812

[35] Wei G.H. , Badis G., Berger M.F., Kivioja T., Palin K., Enge M., Bonke M., Jolma A., Varjosalo M., Gehrke A.R. et al. Genome-wide analysis of ETS-family DNA-binding in vitro and in vivo. EMBO J. 2010; 29:2147–2160. 20517297 10.1038/emboj.2010.106 PMC2905244

[36] Quinlan A.R. , Hall I.M. BEDTools: a flexible suite of utilities for comparing genomic features. Bioinformatics. 2010; 26:841–842. 20110278 10.1093/bioinformatics/btq033 PMC2832824

[37] Sherry S.T. , Ward M.H., Kholodov M., Baker J., Phan L., Smigielski E.M., Sirotkin K. dbSNP: the NCBI database of genetic variation. Nucleic Acids Res. 2001; 29:308–311. 11125122 10.1093/nar/29.1.308 PMC29783

[38] Danecek P. , Auton A., Abecasis G., Albers C.A., Banks E., DePristo M.A., Handsaker R.E., Lunter G., Marth G.T., Sherry S.T. et al. The variant call format and VCFtools. Bioinformatics. 2011; 27:2156–2158. 21653522 10.1093/bioinformatics/btr330 PMC3137218

[39] Meeroff M. History of the Argentinian Society of Gastroenterology (SAGE). Acta Gastroenterol. Latinoam. 1994; 24:195–198. 7701901

[40] Welter D. , MacArthur J., Morales J., Burdett T., Hall P., Junkins H., Klemm A., Flicek P., Manolio T., Hindorff L. et al. The NHGRI GWAS Catalog, a curated resource of SNP-trait associations. Nucleic Acids Res. 2014; 42:D1001–D1006. 24316577 10.1093/nar/gkt1229 PMC3965119

[41] Li M.J. , Liu Z., Wang P., Wong M.P., Nelson M.R., Kocher J.P., Yeager M., Sham P.C., Chanock S.J., Xia Z. et al. GWASdb v2: an update database for human genetic variants identified by genome-wide association studies. Nucleic Acids Res. 2016; 44:D869–D876. 26615194 10.1093/nar/gkv1317 PMC4702921

[42] Gong J. , Mei S., Liu C., Xiang Y., Ye Y., Zhang Z., Feng J., Liu R., Diao L., Guo A.Y. et al. PancanQTL: systematic identification of cis-eQTLs and trans-eQTLs in 33 cancer types. Nucleic Acids Res. 2018; 46:D971–D976. 29036324 10.1093/nar/gkx861 PMC5753226

[43] Xia K. , Shabalin A.A., Huang S., Madar V., Zhou Y.H., Wang W., Zou F., Sun W., Sullivan P.F., Wright F.A. seeQTL: a searchable database for human eQTLs. Bioinformatics. 2012; 28:451–452. 22171328 10.1093/bioinformatics/btr678 PMC3268245

[44] Gamazon E.R. , Zhang W., Konkashbaev A., Duan S., Kistner E.O., Nicolae D.L., Dolan M.E., Cox N.J. SCAN: SNP and copy number annotation. Bioinformatics. 2010; 26:259–262. 19933162 10.1093/bioinformatics/btp644 PMC2852202

[45] Li X. , Shi L., Wang Y., Zhong J., Zhao X., Teng H., Shi X., Yang H., Ruan S., Li M. et al. OncoBase: a platform for decoding regulatory somatic mutations in human cancers. Nucleic Acids Res. 2019; 47:D1044–D1055. 30445567 10.1093/nar/gky1139 PMC6323961

[46] Teng L. , He B., Wang J., Tan K. 4DGenome: a comprehensive database of chromatin interactions. Bioinformatics. 2015; 31:2560–2564. 25788621 10.1093/bioinformatics/btv158 PMC4514924

[47] Wang S. , Sun H., Ma J., Zang C., Wang C., Wang J., Tang Q., Meyer C.A., Zhang Y., Liu X.S. Target analysis by integration of transcriptome and ChIP-seq data with BETA. Nat. Protoc. 2013; 8:2502–2515. 24263090 10.1038/nprot.2013.150 PMC4135175

[48] Huynh-Thu V.A. , Irrthum A., Wehenkel L., Geurts P. Inferring regulatory networks from expression data using tree-based methods. PLoS One. 2010; 5:e12776. 20927193 10.1371/journal.pone.0012776 PMC2946910

[49] Lachmann A. , Giorgi F.M., Lopez G., Califano A. ARACNe-AP: gene network reverse engineering through adaptive partitioning inference of mutual information. Bioinformatics. 2016; 32:2233–2235. 27153652 10.1093/bioinformatics/btw216 PMC4937200

[50] Han H. , Cho J.W., Lee S., Yun A., Kim H., Bae D., Yang S., Kim C.Y., Lee M., Kim E. et al. TRRUST v2: an expanded reference database of human and mouse transcriptional regulatory interactions. Nucleic Acids Res. 2018; 46:D380–D386. 29087512 10.1093/nar/gkx1013 PMC5753191

[51] Corces M.R. , Granja J.M., Shams S., Louie B.H., Seoane J.A., Zhou W., Silva T.C., Groeneveld C., Wong C.K., Cho S.W. et al. The chromatin accessibility landscape of primary human cancers. Science. 2018; 362:eaav1898. 30361341 10.1126/science.aav1898 PMC6408149

[52] Consortium, G.T. The Genotype-Tissue Expression (GTEx) project. Nat. Genet. 2013; 45:580–585. 23715323 10.1038/ng.2653 PMC4010069

[53] Barretina J. , Caponigro G., Stransky N., Venkatesan K., Margolin A.A., Kim S., Wilson C.J., Lehar J., Kryukov G.V., Sonkin D. et al. The Cancer Cell Line Encyclopedia enables predictive modelling of anticancer drug sensitivity. Nature. 2012; 483:603–607. 22460905 10.1038/nature11003 PMC3320027

[54] de Rie D. , Abugessaisa I., Alam T., Arner E., Arner P., Ashoor H., Astrom G., Babina M., Bertin N., Burroughs A.M. et al. An integrated expression atlas of miRNAs and their promoters in human and mouse. Nat. Biotechnol. 2017; 35:872–878. 28829439 10.1038/nbt.3947 PMC5767576

[55] Pinero J. , Ramirez-Anguita J.M., Sauch-Pitarch J., Ronzano F., Centeno E., Sanz F., Furlong L.I. The DisGeNET knowledge platform for disease genomics: 2019 update. Nucleic Acids Res. 2020; 48:D845–D855. 31680165 10.1093/nar/gkz1021 PMC7145631

[56] Becker K.G. , Barnes K.C., Bright T.J., Wang S.A. The genetic association database. Nat. Genet. 2004; 36:431–432. 15118671 10.1038/ng0504-431

[57] Law M. , Shaw D.R. Mouse genome informatics (MGI) Is the international resource for information on the laboratory mouse. Methods Mol. Biol. 2018; 1757:141–161. 29761459 10.1007/978-1-4939-7737-6_7

[58] Oughtred R. , Stark C., Breitkreutz B.J., Rust J., Boucher L., Chang C., Kolas N., O”Donnell L., Leung G., McAdam R. et al. The BioGRID interaction database: 2019 update. Nucleic Acids Res. 2019; 47:D529–D541. 30476227 10.1093/nar/gky1079 PMC6324058

[59] Kanehisa M. , Sato Y., Kawashima M., Furumichi M., Tanabe M. KEGG as a reference resource for gene and protein annotation. Nucleic Acids Res. 2016; 44:D457–D462. 26476454 10.1093/nar/gkv1070 PMC4702792

[60] Jassal B. , Matthews L., Viteri G., Gong C., Lorente P., Fabregat A., Sidiropoulos K., Cook J., Gillespie M., Haw R. et al. The reactome pathway knowledgebase. Nucleic Acids Res. 2020; 48:D498–D503. 31691815 10.1093/nar/gkz1031 PMC7145712

[61] Cerami E.G. , Gross B.E., Demir E., Rodchenkov I., Babur O., Anwar N., Schultz N., Bader G.D., Sander C. Pathway Commons, a web resource for biological pathway data. Nucleic Acids Res. 2011; 39:D685–D690. 21071392 10.1093/nar/gkq1039 PMC3013659

[62] Hanahan D. , Weinberg R.A. Hallmarks of cancer: the next generation. Cell. 2011; 144:646–674. 21376230 10.1016/j.cell.2011.02.013

[63] Gene Ontology, C. The Gene Ontology project in 2008. Nucleic Acids Res. 2008; 36:D440–D444. 17984083 10.1093/nar/gkm883 PMC2238979

[64] Liu W. , Stein P., Cheng X., Yang W., Shao N.Y., Morrisey E.E., Schultz R.M., You J. BRD4 regulates Nanog expression in mouse embryonic stem cells and preimplantation embryos. Cell Death Differ. 2014; 21:1950–1960. 25146928 10.1038/cdd.2014.124 PMC4227152

[65] Gryder B.E. , Yohe M.E., Chou H.C., Zhang X., Marques J., Wachtel M., Schaefer B., Sen N., Song Y., Gualtieri A. et al. PAX3-FOXO1 establishes myogenic super enhancers and confers BET bromodomain vulnerability. Cancer Discov. 2017; 7:884–899. 28446439 10.1158/2159-8290.CD-16-1297 PMC7802885

[66] Gonzales-Cope M. , Sidoli S., Bhanu N.V., Won K.J., Garcia B.A. Histone H4 acetylation and the epigenetic reader Brd4 are critical regulators of pluripotency in embryonic stem cells. BMC Genomics. 2016; 17:95. 26847871 10.1186/s12864-016-2414-y PMC4740988

[67] Wang W. , Tang Y.A., Xiao Q., Lee W.C., Cheng B., Niu Z., Oguz G., Feng M., Lee P.L., Li B. et al. Stromal induction of BRD4 phosphorylation results in chromatin remodeling and BET inhibitor resistance in colorectal cancer. Nat. Commun. 2021; 12:4441. 34290255 10.1038/s41467-021-24687-4 PMC8295257

[68] Donati B. , Lorenzini E., Ciarrocchi A. BRD4 and cancer: going beyond transcriptional regulation. Mol. Cancer. 2018; 17:164. 30466442 10.1186/s12943-018-0915-9 PMC6251205

[69] Ma T. , Hu C., Lal B., Zhou W., Ma Y., Ying M., Prinos P., Quinones-Hinojosa A., Lim M., Laterra J. et al. Reprogramming transcription factors Oct4 and Sox2 Induce a BRD-dependent immunosuppressive transcriptome in GBM-propagating cells. Cancer Res. 2021; 81:2457–2469. 33574085 10.1158/0008-5472.CAN-20-2489 PMC8137560

