# Supplementary material for: TcoFBase: a comprehensive database for decoding the regulatory transcription co-factors in human and mouse
Source: Nucleic Acids Res. 2021 Oct 30;50(D1):D391–401. doi: 10.1093/nar/gkab950 (PMC8728270; doi:10.1093/nar/gkab950)
Supplement: gkab950_Supplemental_Files [file gkab950_supplemental_files.zip › Supplementary Figure S1(1).pdf]

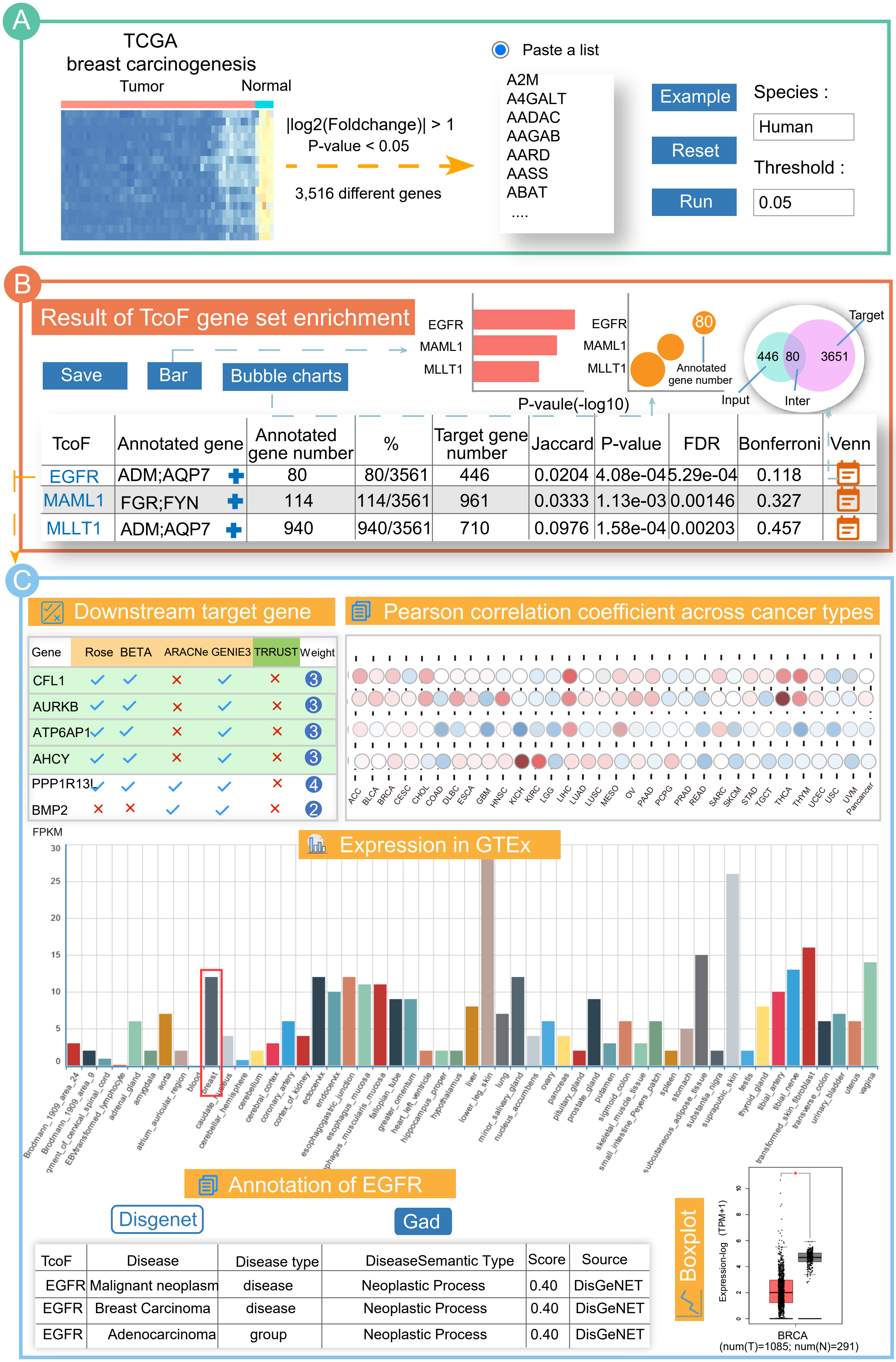
A
TCGA
breast carcinogenesis
Tumor
Normal
|log2(Foldchange)| > 1
P-value < 0.05
3,516 different genes
Paste a list
A2M
A4GALT
AADAC
AAGAB
AARD
AASS
ABAT
....
Example
Reset
Run
Species :
Human
Threshold :
0.05
B
Result of TcoF gene set enrichment
Save
Bar
Bubble charts
EGFR
MAML1
MLLT1
P-vaule(-log10)
80
Annotated gene number
Target
446
80
3651
Input
Inter
TcoF | Annotated gene | Annotated gene number | % | Target gene number | Jaccard | P-value | FDR | Bonferroni | Venn
EGFR | ADM;AQP7 | 80 | 80/3561 | 446 | 0.0204 | 4.08e-04 | 5.29e-04 | 0.118
MAML1 | FGR;FYN | 114 | 114/3561 | 961 | 0.0333 | 1.13e-03 | 0.00146 | 0.327
MLLT1 | ADM;AQP7 | 940 | 940/3561 | 710 | 0.0976 | 1.58e-04 | 0.00203 | 0.457
C
Downstream target gene
Gene | Rose | BETA | ARACNe | GENIE3 | TRRUST | Weight
CFL1 | ✓ | ✓ | ✗ | ✓ | ✗ | 3
AURKB | ✓ | ✓ | ✗ | ✓ | ✗ | 3
ATP6AP1 | ✓ | ✓ | ✗ | ✓ | ✗ | 3
AHCY | ✓ | ✓ | ✗ | ✓ | ✗ | 3
PPP1R13L | ✓ | ✓ | ✓ | ✓ | ✗ | 4
BMP2 | ✗ | ✗ | ✓ | ✓ | ✗ | 2
Pearson correlation coefficient across cancer types
ACC BLCA BRCA CESC CHOL COAD DLBC ESCA GBM HNSC KICH KIRC LGG LIHC LUAD LUSC MESO OV PAAD PCPG PRAD READ SARC SKCM STAD TGCT THCA THYM UCEC USC UVM Pancancer
Expression in GTEx
FPKM
30
25
20
15
10
5
0
Brodmann_1909_area_24
Brodmann_1909_area_9
gment_of_cervical_spinal_cord
EBVtransformed_lymphocyte
adrenal_gland
amygdala
aorta
atrium_auricular_region
blood
breast
caudate
uterus
cerebellar_hemisphere
cerebellum
cerebral_cortex
coronary_artery
cortex_of_kidney
ectocervix
endocervix
esophagogastric_junction
esophagus_mucosa
phagus_muscularis_mucosa
fallopian_tube
greater_omentum
heart_left_ventricle
hippocampus_proper
hypothalamus
liver
lower_leg_skin
lung
minor_salivary_gland
nucleus_accumbens
ovary
pancreas
pituitary_gland
prostate_gland
putamen
sigmoid_colon
skeletal_muscle_tissue
small_intestine_Peyers_patch
spleen
stomach
subcutaneous_adipose_tissue
substantia_nigra
suprapubic_skin
testis
thyroid_gland
tibial_artery
tibial_nerve
transformed_skin_fibroblast
transverse_colon
urinary_bladder
uterus
vagina
Annotation of EGFR
Disgenet
Gad
TcoF | Disease | Disease type | DiseaseSemantic Type | Score | Source
EGFR | Malignant neoplasm | disease | Neoplastic Process | 0.40 | DisGeNET
EGFR | Breast Carcinoma | disease | Neoplastic Process | 0.40 | DisGeNET
EGFR | Adenocarcinoma | group | Neoplastic Process | 0.40 | DisGeNET
Boxplot
Expression-log (TPM+1)
BRCA
(num(T)=1085; num(N)=291)
