# Supplementary material for: TcoFBase: a comprehensive database for decoding the regulatory transcription co-factors in human and mouse
Source: Nucleic Acids Res. 2021 Oct 30;50(D1):D391–401. doi: 10.1093/nar/gkab950 (PMC8728270; doi:10.1093/nar/gkab950)
Supplement: gkab950_Supplemental_Files [file gkab950_supplemental_files.zip › Supplementary Figure S2.pdf]

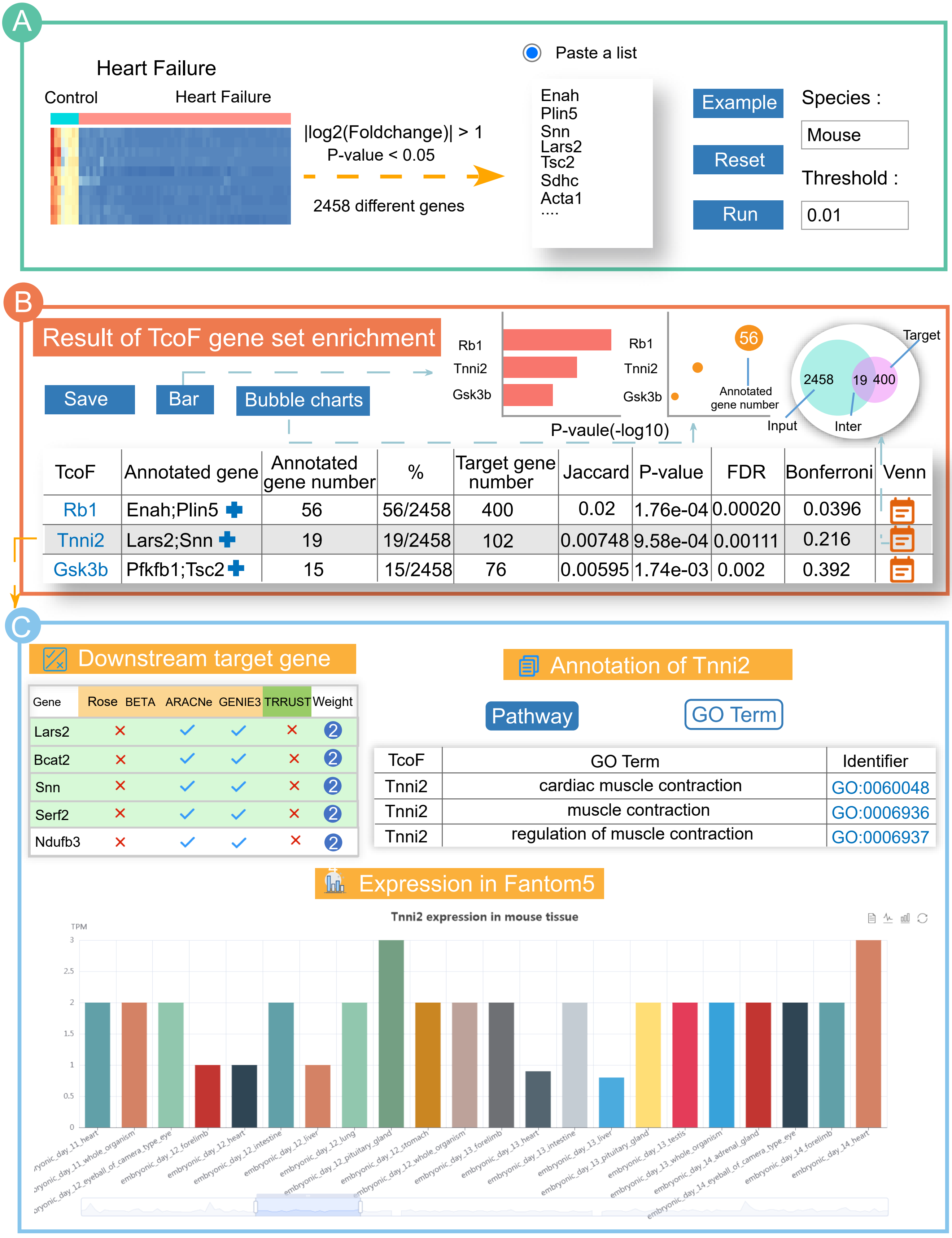
A
Heart Failure
Control
Heart Failure
|log2(Foldchange)| > 1
P-value < 0.05
2458 different genes
Paste a list
Enah
Plin5
Snn
Lars2
Tsc2
Sdhc
Acta1
....
Example
Reset
Run
Species :
Mouse
Threshold :
0.01
B
Result of TcoF gene set enrichment
Save
Bar
Bubble charts
Rb1
Tnni2
Gsk3b
P-vaule(-log10)
56
Annotated gene number
Target
2458
19
400
Input
Inter
TcoF
Annotated gene
Annotated gene number
%
Target gene number
Jaccard
P-value
FDR
Bonferroni
Venn
Rb1 Enah;Plin5 56 56/2458 400 0.02 1.76e-04 0.00020 0.0396
Tnni2 Lars2;Snn 19 19/2458 102 0.00748 9.58e-04 0.00111 0.216
Gsk3b Pfkfb1;Tsc2 15 15/2458 76 0.00595 1.74e-03 0.002 0.392
C
Downstream target gene
Gene
Rose
BETA
ARACNe
GENIE3
TRRUST
Weight
Lars2
Bcat2
Snn
Serf2
Ndufb3
2
Annotation of Tnni2
Pathway
GO Term
TcoF
GO Term
Identifier
Tnni2 cardiac muscle contraction GO:0060048
Tnni2 muscle contraction GO:0006936
Tnni2 regulation of muscle contraction GO:0006937
Expression in Fantom5
Tnni2 expression in mouse tissue
TPM
3
2.5
2
1.5
1
0.5
0
embryonic_day_12_forelimb
embryonic_day_12_heart
embryonic_day_12_intestine
embryonic_day_12_liver
embryonic_day_12_lung
embryonic_day_12_pituitary_gland
embryonic_day_12_stomach
embryonic_day_12_whole_organism
embryonic_day_13_forelimb
embryonic_day_13_heart
embryonic_day_13_intestine
embryonic_day_13_liver
embryonic_day_13_pituitary_gland
embryonic_day_13_testis
embryonic_day_13_whole_organism
embryonic_day_14_adrenal_gland
embryonic_day_14_eyeball_of_camera_type_eye
embryonic_day_14_forelimb
embryonic_day_14_heart
