# Supplementary material for: TcoFBase: a comprehensive database for decoding the regulatory transcription co-factors in human and mouse
Source: Nucleic Acids Res. 2021 Oct 30;50(D1):D391–401. doi: 10.1093/nar/gkab950 (PMC8728270; doi:10.1093/nar/gkab950)
Supplement: gkab950_Supplemental_Files [file gkab950_supplemental_files.zip › Supplementary Materials.docx]

**Supplementary material of TcoFBase: a comprehensive database for decoding the regulatory transcription co-factors in human and mouse**

**Case study of online analysis**

To illustrate how to use TcoFBase, we used the ‘TcoF gene set enrichment’ analysis to identify the regulatory TcoFs of gene set of interest. Firstly, we obtained 3,561 differentially expressed genes (log2FC > 1, P-adj < 0.05) of breast invasive carcinoma from the TCGA project as inputs for TcoFBase. Next, we set the options, including Species = Human and the hypergeometric test P-value = 0.05, and clicked the ‘Analyze’ button to complete the gene set enrichment analysis (Supplementary Figure 1A). The output table displayed 13 TcoFs were significantly enriched. TcoFBase also provided the bubble, bar graphs and Venn diagram for visualization of the enrichment analysis result. The detailed gene annotations and enrichment analysis results were shown in Supplementary Table 2.

We found that EGFR was significantly enriched from the enrichment analysis result (ranked first of all TcoFs with enrichment P-value = 0.000408), which plays an important role in breast carcinogenesis. Previous study has demonstrated that overexpression of EGFR may promote breast tumorigenesis. The expression of EGFR was an important prognostic factor for patient's overall survival and EGFR also used as a treatment target for breast cancer. These studies revealed the biological relevance of EGFR in breast cancer ([1](#_ENREF_1),[2](#_ENREF_2)).

After clicking ‘EGFR’, the detail page showed the detailed information and function annotations of EGFR. We found that EGFR was highly correlated with breast cancer (i.e., Malignant neoplasm of breast and Breast Carcinoma), which was consistent with the above results (Supplementary Figure 1C). In addition, these TcoFs were significantly enriched in breast cancer, such as MAML1 (ranked second of all TcoFs with enrichment P-value = 0.00113) and ERCC3 (ranked fifth of all TcoFs with enrichment P-value = 0.00868), which were play important roles in breast cancer ([3](#_ENREF_3),[4](#_ENREF_4)).

Several novel TcoFs with high enrichment scores were also enriched in above analysis. For example, MLLT1 has not been widely studied in breast cancer (ranked third of all TcoFs with enrichment P-value = 0.00158). MLLT1 was demonstrated to participate tumorigenesis by controlling transcriptional regulation, suggesting that MLLT1 might be potential novel TcoFs of breast cancer. These results of enrichment analyses can be observed in Supplementary Table 2.

We also performed the same tests for heart failure in mouse and obtained similar results (Supplementary Table 2). We found that Tnni2 is critical for muscular system development and dysfunction of Tnni2 will lead to myocardial abnormalities and heart failure. After clicking ‘Tnni2’, the detail page exhibited the detailed annotation information of Tnni2. In the section of the ‘Annotation’, Tnni2 was associated with gene ontology of [cardiac muscle contraction](https://www.ebi.ac.uk/QuickGO/term/GO:0060048) and regulation of muscle contraction, which was consistent with our expectations (Supplementary Figure 2C). The results demonstrate the availability and biological value of TcoFBase in TcoFs research.

**REFERENCES**

1. Hwangbo, W., Lee, J.H., Ahn, S., Kim, S., Park, K.H., Kim, C.H. and Kim, I. (2013) EGFR Gene Amplification and Protein Expression in Invasive Ductal Carcinoma of the Breast. *Korean journal of pathology*, **47**, 107-115.

2. Park, K., Han, S., Shin, E., Kim, H.J. and Kim, J.Y. (2007) EGFR gene and protein expression in breast cancers. *European journal of surgical oncology : the journal of the European Society of Surgical Oncology and the British Association of Surgical Oncology*, **33**, 956-960.

3. Shi, W., Tang, T., Li, X., Deng, S., Li, R., Wang, Y., Wang, Y., Xia, T., Zhang, Y., Zen, K. *et al.* (2019) Methylation-mediated silencing of miR-133a-3p promotes breast cancer cell migration and stemness via miR-133a-3p/MAML1/DNMT3A positive feedback loop. *Journal of experimental & clinical cancer research : CR*, **38**, 429.

4. Vijai, J., Topka, S., Villano, D., Ravichandran, V., Maxwell, K.N., Maria, A., Thomas, T., Gaddam, P., Lincoln, A., Kazzaz, S. *et al.* (2016) A Recurrent ERCC3 Truncating Mutation Confers Moderate Risk for Breast Cancer. *Cancer discovery*, **6**, 1267-1275.

**Supplementary Materials.** Case study of online analysis.

**Supplementary Table 1.** The statistics about the content of TcoFBase.

**Supplementary Table 2.** The enrichment analysis results of BRCA.

**Supplementary Table 3.** The enrichment analysis results of Heart failure.

**Supplementary Figure 1.** Results of enrichment analyses associated with 3,561 differential expressed genes in BRCA. (A) Input and parameter selection for the enrichment analysis. (B) Results table for enrichment analysis sand validation results for enrichment analysis. (C) Detailed results for “EGFR”. The annotated genes are shown in green in downstream target genes.

**Supplementary Figure 2**. Results of enrichment analyses associated with 2,458 differentially expressed genes in heart failure. (A) Input and parameter selection for the enrichment analysis. (B) Results table for enrichment analysis sand validation results for enrichment analysis. (C) Detailed results for “Tnni2”. The annotated genes are shown by green in downstream target genes.
