# Supplementary material for: TcoFBase: a comprehensive database for decoding the regulatory transcription co-factors in human and mouse
Source: Nucleic Acids Res. 2021 Oct 30;50(D1):D391–401. doi: 10.1093/nar/gkab950 (PMC8728270; doi:10.1093/nar/gkab950)
Supplement: gkab950_Supplemental_Files [file gkab950_supplemental_files.zip › Supplementary Table1.docx]

**Supplementary Table 1.** The statistics about the content of TcoFBase

| **Element** | **Source** | **Human** | **Mouse** |
| --- | --- | --- | --- |
| TcoF | 2 | 1,244 | 1,078 |
| TcoF-associated ChIP-seq sample | 4 | 4,246 | 2,513 |
| Super-enhancer | 3 | 2,678,273 | 11,609 |
| Enhancer | 6 | 14,797,266 | 439,092 |
| TFBS | 1 | 5,547,656 | 2,858,356 |
| Motif | 6 | 3,279 | 3,279 |
| Common SNP | 1 | 38,063,729 | -- |
| Risk SNP | 2 | 264,514 | -- |
| eQTL | 4 | 2,886,133 | -- |
| DHS | 2 | 69,860,705 | 9,802,229 |
| 450K | 1 | 30,392,523 | -- |
| WGBS | 1 | 166,855,665 | -- |
| Chromatin interaction | 2 | 29,137,183 | 4,336,844 |
